# Combination Vaccination With Tetanus Toxoid and Enhanced Tumor-Cell Based Vaccine Against Cervical Cancer in a Mouse Model

**DOI:** 10.3389/fimmu.2020.00927

**Published:** 2020-05-27

**Authors:** Donia Alson, Scott C. Schuyler, Bo-Xin Yan, Karthika Samimuthu, Jiantai Timothy Qiu

**Affiliations:** ^1^Graduate Institute of Biomedical Sciences, College of Medicine, Chang Gung University, Taoyuan, Taiwan; ^2^Department of Biomedical Sciences, Chang Gung University, Taoyuan, Taiwan; ^3^Division of Head & Neck Surgery, Department of Otolaryngology, Chang Gung Memorial Hospital, Taoyuan, Taiwan; ^4^Department of Obstetrics and Gynecology, Chang Gung Memorial Hospital, Taoyuan, Taiwan; ^5^Department of Obstetrics and Gynecology, Taipei Medical University Hospital, Taipei, Taiwan

**Keywords:** human papillomavirus, vaccination, granulocyte macrophage-colony stimulating factor, cervical cancer, tetanus toxoid

## Abstract

Cervical cancer is the fourth most common cancer in women with an estimated 570,000 new cases in 2018 which constitute about 6. 6% of all cancers in women according to WHO report 2018. Approximately 90% of the 270,000 deaths from cervical cancer in 2015 occurred in low- and middle-income countries. In cervical cancers, which is caused by human papillomavirus (HPV) infection, the expression of HPV 16 E6 and E7 proteins are essential for tumor cell transformation and maintenance of malignancy. Prophylactic vaccines against cervical cancer caused by human papillomavirus have not proven successful. Although virus-like particle-based (VLPs) vaccines have been developed with prophylactic activities to prevent most HPV infections, the therapeutic effect of VLP vaccines has yet to be demonstrated for those who were already infected. A recent study showed that pre-conditioning mice with a potent antigen such as tetanus toxoid significantly improves lymph node homing and efficacy of dendritic cells. Tetanus toxoid has also been used in combination with DNA vaccines designed from tumor based antigens. In the present study, we pre-conditioned mice with tetanus toxoid followed by vaccination with a Granulocyte-Macrophage Colony-Stimulating Factor (GM-CSF) overexpressing tumor-cell based vaccine (GVAX). We observed that pre-conditioning with tetanus toxoid followed by vaccination with GVAX regressed tumor growth and enhanced the overall survival of the mice. Pre-conditioning with tetanus toxoid enhanced the immune response which was observed by enlarged spleen size, higher proliferation rate of lymphocytes, a higher level of IFN-γ, TNF-α, and IL-4 antigen-specific secretions by the splenocytes. Pre-conditioning with tetanus toxoid increased memory T cell migration into the tumor site and spleen. The antigen-specific cytotoxic T cell lysis percentage was also found to be higher in the group of mice vaccinated with the combination of tetanus toxoid and GVAX. Hence, pre-conditioning with tetanus toxoid prior to vaccination with a tumor-cell based vaccine overexpressing GM-CSF might be an effective strategy for targeting E7-specific HPV-associated cervical malignancy.

## Introduction

Cervical cancer is the fourth most common malignancy in women with an estimated 260,000 deaths every year worldwide (1). Human papilloma virus (HPV) types 16 and 18 account for roughly 70% of all cervical cancer cases (2, 3). The HPV early proteins E6 and E7 are the main mediators for the induction of HPV-associated cervical cancer as both the proteins are consistently expressed in HPV-associated cervical cancer cells (4, 5). E6 inactivates the tumor suppressor p53 by binding to the ubiquitin ligase E6AP and inhibits the p53-mediated signaling pathway (6–9), whereas E7 binds with pRb and promotes its proteasomal degradation (10–12). Since E6 and E7 proteins are constitutively expressed in HPV-associated cervical cancer cells, these tumor-specific antigens serve as an attractive therapeutic strategy to target HPV-associated cervical cancer cells.

Polyvalent vaccines, such as irradiated whole-cell vaccines, can activate T cells and cells of the innate immune system such as natural killer cells, macrophages, and eosinophils (12–14). The advantage of using a whole-cell vaccine, rather than a single peptide or protein as a vaccine, is that whole-cell vaccines provide all potential antigens present in the tumor cell (15–17). Whole-cell vaccines were also developed by genetically modifying the cells that express cytokines to induce an elevated immune response to the injected irradiated tumor cells (17–19). Currently available prophylactic vaccines developed from HPV virus-like particles (VLPs) against HPV-associated cervical cancer are successful in preventing tumor growth but are unsuccessful in eradicating the already infected cells (20, 21). Although prophylactic vaccines are successful in generating antibodies, a cellular immune response against E6 and E7 is required to eliminate already infected cells (22, 23). Hence, the development of new vaccine approaches is necessary to eradicate cells already infected with HPV.

Bacteria and bacterial antigens have been used in cancer immunotherapy research for many years. Coley's toxin, which consists of a mixture of inactivated *Streptococcus pyogens* and *Serratia marcescens* showed promising results by inducing tumor regression (24–26). A recent study showed that preconditioning mice and patients with bacterial antigens such as tetanus toxoid followed by vaccination with tumor antigen-specific vaccines enhanced dendritic cell migration and overall survival of both mice and humans (27). These results indicate the significance of using bacterial antigens to enhance the immune response and for promoting the regress of tumor growth.

In the present study we evaluated the immune response of mice vaccinated with tetanus toxoid and irradiated TC-1 cells, a model cell for HPV driven tumorigenesis, by engineering the TC-1 cells to secrete codon-optimized GM-CSF (GVAX). We assessed the efficacy of tetanus toxoid and tumor cell-based combination vaccination to suppress tumor growth and assessed the overall survival of the mice. Our results showed that combination vaccination with tetanus toxoid and GVAX regressed tumor growth via the Th1 and Th2 cell cytokine responses and increased the overall survival of the mice. In addition, the combination vaccination induced a higher percentage of memory T cells and elevated the generation of cytotoxic effector T cells *in vivo*.

## Materials and Methods

### Mice

Female C57BL/6 (B6) mice were purchased from the National Laboratory Animal Center (Taipei, Taiwan) and housed under specific pathogen-free conditions at the animal facility of Chang Gung University. All the mice used for the experiments were of an age between 8 and 12 weeks and were kept in individually ventilated cages. All the experiments were performed in accordance with the Animal Experimental Ethics Committee of Chang Gung University. The statement of approval from the Ethics Committee contains the ethical code “CGU107-288” with the date 1st August, 2019.

### Cell Lines

The TC-1 cells were engineered by the transformation of primary C57BL/6 mouse lung epithelial cells with HPV type-16, E6/E7 oncogenes and an activated *H-ras* oncogene, as described previously (28). Stable TC-1 cells expressing wild-type GM-CSF (wt-GM-CSF) or codon-optimized GM-CSF (cGM-CSF) (GVAX) were established by lentiviral infection of TC-1 cell lines as previously described (29–31). The cell lines were maintained in RPMI 1640 medium (Gibco, Waltham, MA, USA) supplemented with 2 mM L-glutamine, 25 mM HEPES, 24 mM sodium biocarbonate, 10% heat-inactivated fetal bovine serum (Invitrogen, Waltham, MA, USA), 100 U/mL penicillin, 100 mg/mL streptomycin, and 50 μM β-mercaptoethanol at 37°C in an atmosphere of 5% CO_2_.

### Tumor Model and Vaccination

For the *in vivo* tumor protection experiments, C57BL/6 mice (*n* = 5 per group) were immunized intramuscularly with 1Lf, 100 μl tetanus toxoid (Kuo Kwang, Taichung, Taiwan) into the quadriceps muscle of each mouse. Two weeks later, a booster dose was given intramuscularly. Seven days after the booster vaccination, the mice were immunized subcutaneously in the dorsal flank with 4 × 10^6^ irradiated (10,000 cGy) TC-1/cGM-CSF cells. Two weeks later, a booster dose of TC-1/cGM-CSF cells was given. Seven days after the final vaccination, the immunized mice were subcutaneously challenged with 2 × 10^5^ TC-1 cells in the right dorsal flank. Tumor growth was monitored three times a week using calipers and tumor volume was calculated using the formula: Length x (width)^2^ × 0.5. When the tumor growth exceeded 2 cm in diameter, the mice were considered dead from the tumor burden and were subsequently euthanized (Figure A1).

### Spleen Weight Index and Splenocyte Proliferation

The spleens and lymph nodes from immunized mice were aseptically harvested, transferred to six-well culture plate containing RPMI 1640 medium supplemented with 10% fetal bovine serum and penicillin (10 U/mL) and weighted. Spleen indices were calculated as organ weight (milligram, mg) per gram of mouse body weight. The splenocytes (5 × 10^5^ cells/well) were incubated with 10 μg/mL of E7 peptide or medium alone (unstimulated) in triplicate for 72 h at 37°C. The proliferation activity was examined by a CFSE cell proliferation assay kit (Thermo Fisher Scientific, Waltham, MA, USA).

### Cytokine Secretion Measurement With ELISA

GM-CSF, IFN-γ, TNF-α, and IL-4 levels from the secreting splenocytes were measured by ELISA with a commercially available ELISA kit (Biolegend, San Diego, CA, USA). A total of 1 × 10^6^ TC-1 cells were cultured in 7 mL of the medium for 24 h in a 10 cm dish for GM-CSF analyses. Supernatants were collected by centrifugation at 220x rcf for 4 min and diluted appropriately for the assay. To analyze IFN-γ, TNF-α, and IL-4, splenocytes were harvested from the mice, stimulated with E7 peptide for 72 h. Standards or experimental samples were added to microtiter plates coated with monoclonal antibody to the cytokine of interest and incubated for 2 h with shaking. After washing, horseradish peroxidase-conjugated, cytokine specific antibody was added to each well and incubated for 1 h and washed. Substrate solution was added and incubated for 30 min with shaking and the reaction was stopped using stop solution. Cytokine levels were determined by measuring the optical density at 450 nm by using microtiter plate reader (Molecular Devices, San Jose, CA, USA).

### Flow Cytometric Analyses of Immune Cells

For memory T cell analyses, splenocytes from various vaccinated groups were harvested, lysed with 1x RBC lysis buffer, washed and 1 × 10^6^ cells per well was utilized for each reaction followed by flow cytometry (BD FACSCaliber, Hampton, NH, USA) analysis. Splenocytes were stained with anti-CD44-APC (Biolegend, San Diego, CA, USA), anti-CD4-FITC (Biolegend, San Diego, CA, USA), anti-CD8-PE (Biolegend, San Diego, CA, USA), anti-CD25-APC (Biolegend, San Diego, CA, USA), or anti-Foxp3-APC (Biolegend, San Diego, CA, USA).

### *In vivo* Cytotoxicity Assays

A total of 10^7^ spleen cells from naïve syngeneic spleen cells from wild type C57BL/6 mice used as *in vivo* target cells. Cells were labeled with either 5 M carboxyflourescein succinimidyl ester (CFSE^high^) or 0.5 M CFSE (CFSE^low^) for 15 min at 37°C and washed twice with phosphate-buffered saline (PBS). CFSE^high^ cells were incubated with MHC Class-I restricted E7 peptide (10 μg/mL) or MHC Class-II restricted E7 peptide (10 μg/mL) in CTL medium for 1 h at 37°C. CFSE^low^ cells were incubated with CTL medium without peptide, serving as an internal control. CFSE labeled cells were then washed twice with PBS. A mixture of 2.5 × 10^7^ CFSE^high^ and CFSE^low^ cells were injected intravenously through the tail vein. After 12 h of *in vivo* incubation, splenocytes were harvested and single cell suspensions were analyzed for detection and quantification of CFSE-labeled cells. The percent specific lysis was determined by the ratio of recovered non-peptide-treated control spleen cells to peptide-sensitized spleen cells (percentage of CFSE^low^ cells/percentage of CFSE^high^ cells).

### Statistical Analyses

All the analyses were performed using GraphPad Prism statistical software (Graph Pad Software, La Jolla, CA, USA). One-way ANOVA and log-rank (Mantel-Cox) tests were used to analyze the tumor growth and mouse survival data, respectively. A value of ^*^p < 0.05, ^**^p < 0.01, ^***^p < 0.001 were considered statistically significant.

## Results

### Higher Levels of GM-CSF Secretion by TC-1/cGM-CSF Stable Cell Lines

**Figure 1:**
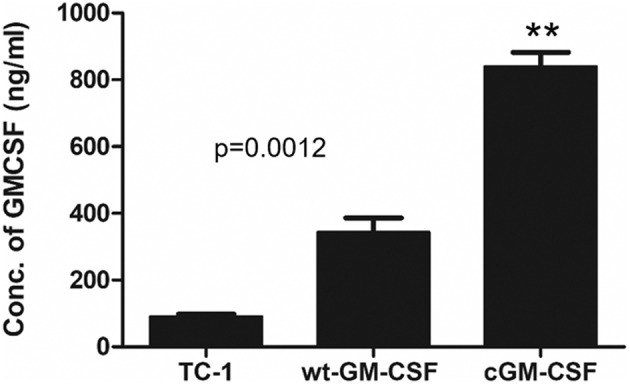
Increased levels of GM-CSF secretion by TC-1 cells containing codon-modified GM-CSF (cGM-CSF). The level of GM-CSF production was quantified by performing ELISA. The results shown are from three independent experiments. **p < 0.01; single classification ANOVA.

To check whether stable cell lines can enhance the level of GM-CSF compared with other cell lines such as TC-1 cells and TC-1 cell expressing wild-type GM-CSF (TC-1/wt-GM-CSF) we measured the levels of protein using TC-1 cell expressing codon-optimized GM-CSF (TC-1/cGM-CSF) by ELISA. Cells were grown in culture media, and medium containing GM-CSF was collected from the cells cultured for 24 h to perform ELISA. As shown in Figure 1, TC-1 cells infected with lentivirus cGM-CSF produced increased levels of GM-CSF compared with the TC-1 cells infected with lentivirus wt-GM-CSF. These results show that GM-CSF is expressed more effectively when its codons are optimized.

### Vaccination With Tetanus Toxoid and GVAX Induced Enhanced Splenocyte Proliferation

**Figure 2:**
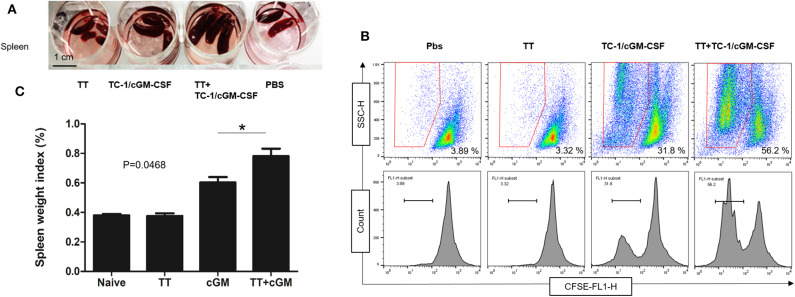
Enhanced splenocyte proliferation after combination vaccination. C57BL/6 mice (*n* = 5) were injected with tetanus toxoid or irradiated TC-1/cGM-CSF cells (GVAX) or combination of both (4 × 10^6^) by intramuscular and subcutaneously injections at the dorsal flank. Seven days after the last immunization, the spleens from the mice were harvested weighed and analyzed for splenocyte proliferation. **(A)** The spleens from immunized mice were isolated and weighed. **(B)** Spleen weight index was calculated as organ weight per gram of mouse body weight. **(C)** The proliferation activity of splenic lymphocytes was measured under stimulation with E7 peptide. *p < 0.05; single classification ANOVA.

Seven days after the final vaccination, spleens were isolated and weighed (Figure A1). As shown in the Figures 2A,B mice vaccinated with combination of tetanus toxoid and TC-1/cGM-CSF (GVAX) had much larger spleen weight indices compared with the mice vaccinated with either tetanus toxoid or GVAX alone. The spleen weight index is presented as the ratio of spleen weight to the mouse body weight. This indicates that combination vaccination with tetanus toxoid and GVAX might induce higher levels of splenocytes proliferation. We then evaluated the E7_49−57_ peptide specific proliferation under stimulation with the E7_49−57_ peptide employing a CFSE cell proliferation assay, and found that the splenocytes from the group of mice that had received the combination vaccination of tetanus toxoid and GVAX (56.2%) showed significantly higher proliferative activity than that from the GVAX alone (31.8%) group or tetanus toxoid alone group (3.32%). The lymphocytes from PBS group showed no obvious proliferation activity against the E7_49−57_ peptide. This result shows that the spleen weight index was positively correlated with the splenocyte proliferation percentage.

### Increased Levels of Th1 Cell and Th2 Cell Cytokines in Mice Vaccinated With the Combination Vaccination

**Figure 3:**
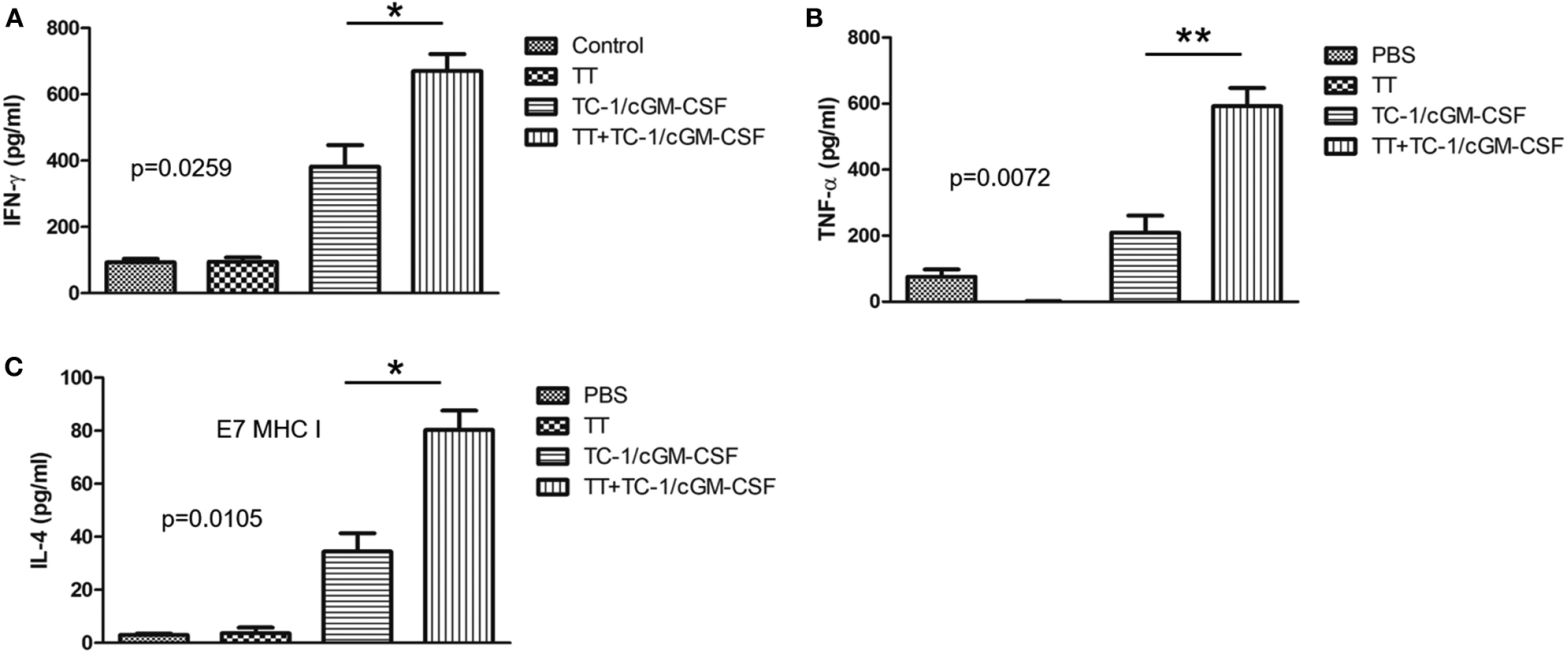
Combination of tetanus toxoid and GVAX vaccination enhances the level of Th1 cell and Th2 cell cytokine secretions. C57BL/6 mice (*n* = 5) were injected with tetanus toxoid or irradiated TC-1/cGM-CSF cells or combination of both (4 × 10^6^) by intramuscular and subcutaneously injections at the dorsal flank. Seven days after the last immunization, the spleens from the mice were harvested, cultured with E7 peptide and analyzed for Th1 and Th2 cytokine secretion. The results depict the stimulated secretion of **(A)** IFN-γ, **(B)** TNF-α, and **(C)** IL-4, by splenocytes. The data are presented as the mean ± SD of triplicates. *p < 0.05; **p < 0.01; single-classification ANOVA.

Th1 cell and Th2 cell immunity is critically important for the induction of anti-tumor cellular immunity (32, 33). Th1 cells secrete cytokines such as IFN-γ, TNF-α/β, IL-2, and IL-10 (34). Th2 cells secrete different types of cytokines such as IL-10, IL-4, IL-5, and IL-6 (35, 36). To determine whether combination vaccination influences Th1 and Th2 cytokine production, we have analyzed the levels of IFN-γ, TNF-α, and IL-4 responses in the culture supernatants of isolated cells from spleens which were stimulated with HPV 16 E7_49−57_ peptide for 72 h. ELISA was performed to analyze the medium collected from cultured splenocytes (Figure A1). As shown in the Figures 3A–C, there was a significant increase in the levels of IFN-γ (mean = 670 pg/mL) TNF-α (mean = 592 pg/mL), and IL-4 levels (mean = 79 pg/mL) secreted by the splenocytes after 72 h of peptide induction in the group of mice vaccinated with combination vaccination of tetanus toxoid and GVAX compared with the group of mice vaccinated with either tetanus toxoid (IFN-γ mean = 95.32 pg/mL, TNF-α mean = 0.39 pg/mL, IL-4 mean = 5 pg/mL) or GVAX (IFN-γ mean = 381.59 pg/mL, TNF-α mean = 209 pg/mL, IL-4 mean = 38 pg/mL) alone. There was no significant difference in the level of secretion of cytokines such as IFN-γ, TNF-α and IL-4 in the group of mice either vaccinated with PBS or with tetanus toxoid.

### Higher Percentage of Memory T Cell Generation in the Spleen After Combination Vaccination of Tetanus and GVAX

**Figure 4:**
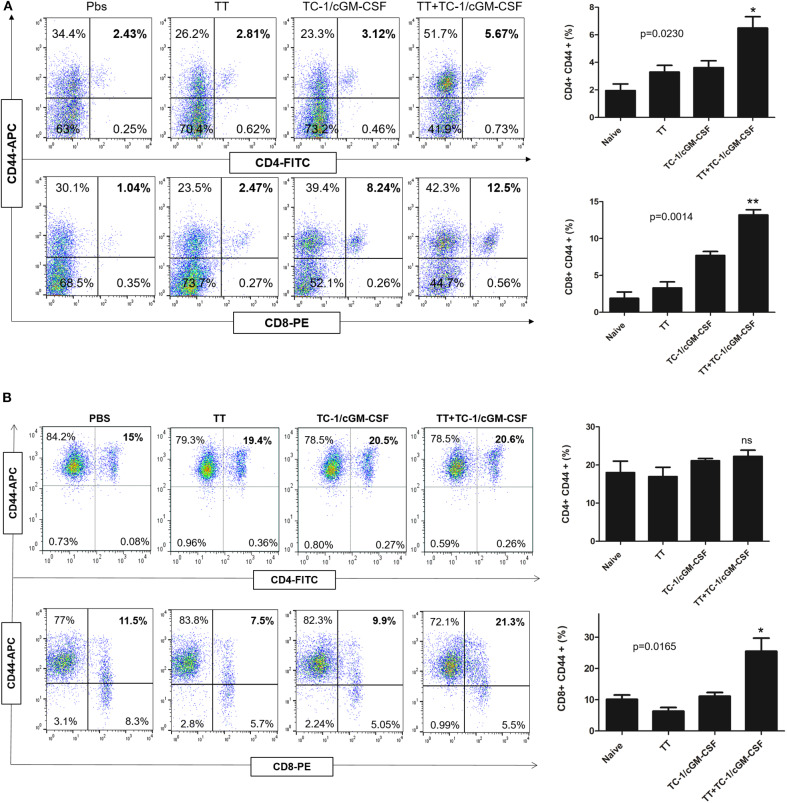
Combination vaccination increased the percentage of CD8+ CD44+ memory T cell lymphocyte in TILs and spleen. C57BL/6 mice (*n* = 5) were injected with tetanus toxoid or GVAX or combination of both (4 × 10^6^) by intramuscular and subcutaneously injections at the dorsal flank. Seven days after the last immunization, the mice were inoculated with 2 × 10^5^ TC-1 cells subcutaneously at the right flank. 30 days after the tumor inoculation, the tumor tissues and spleens were harvested for analysis. **(A)** Representative flow cytometry plots of percentages of CD4+ CD44+ and CD8+ CD44+ memory T cells in the TILs. The bar graph shows the percentage of indicated population of total CD4+ CD8+ CD44+ memory T cells. **(B)** Representative flow cytometry plots of percentages of CD4+ CD44+ and CD8+ CD44+ memory T cells in the spleen. The bar graph shows the percentage of indicated population of total CD4+ CD8+ CD44+ memory T cells. The data are presented as the mean ± SD of duplicate values. *p < 0.05; **p < 0.01; single-classification ANOVA.

Activation of both CD4+ and CD8+ T lymphocytes is important for an efficient immune response to destroy tumor cells (37, 38). CD44 expression is upregulated in naïve T cells after their activation. Memory T cells have intermediate to high expression of CD44. We initially analyzed the mechanism of action of the combination vaccination by measuring the generation of CD44+ memory T cells in the tumor-infiltrating lymphocyte (TIL) population. Thirty days after the inoculation with live TC-1 tumor cells subcutaneously, TILs were harvested from the dissected tumor samples (Figure A1). As shown in Figures 4A,B we detected a higher percentage of memory T cells double positive for CD8 and CD44 T surface markers in the group of mice vaccinated with both tetanus toxoid and GVAX vaccination (12.5%) compared with the group of mice vaccinated with either tetanus toxoid (2.47%) or GVAX (8.24%) alone. Similarly, we also detected a higher percentage of CD4+ and CD44+ double positive memory T cell population (5.67%) in the group of mice vaccinated with the combination vaccination of tetanus toxoid and GVAX (Figure 4A). We also analyzed the spleens and detected a higher percentage of CD8+ and CD44+ double positive memory T cell population (5.67%) in the group of mice vaccinated with the combination vaccination of tetanus toxoid and GVAX (Figure 4B).

### Generation of Cytotoxic Effector T Cells *in vivo* After Combination Vaccination With Tetanus Toxoid and GVAX

**Figure 5:**
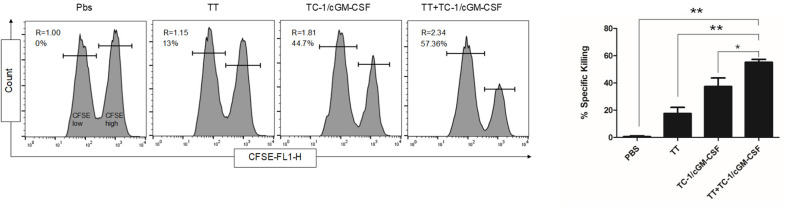
Effects of combination vaccination on the generation E7-specific CTL in C56BL/6 mice. Mice were administered intramuscularly with 1Lf of the tetanus toxoid with a two-week interval of time followed by subcutaneous injection with GVAX twice with one-week time interval. To analyze E7-specific cytotoxicity, cells pooled from the spleen of naïve mice were pulsed with E7 [aa-49-57, RAHYNIVTF] peptide and labeled with a high concentration of CFSE (CFSE^high^) and low concentration of CFSE (CFSE^low^). A 1:1 mixture of each target cell population was injected into recipient mice and specific cytotoxicity was determined 14 h later. Representative histograms of the spleen cells of the mice in each group are shown with percentages of the specific killing of E7 [aa-49-57, RAHYNIVTF] peptide-pulsed target cells in the spleens. The experiments were repeated two times with five animals per group. The data are presented as mean ± SD of duplicate values. *p < 0.05; **p < 0.01; single-classification ANOVA.

We next evaluated the ability of tetanus toxoid and GVAX combination vaccine to generate E7_49−57_ specific cell CTL response in an *in vivo* assay. Anti-tumor function of CD8+ T cells depends on the generation of effector T cells. Seven days after the final vaccination, mice were injected intravenously with a combination of peptide-pulsed (CFSE^high^) and peptide-unpulsed (CFSE^low^) splenocytes and the spleens were harvested after 14 h of *in vivo* incubation. As shown in Figure 5, E7_49−57_ specific lysis was detected in TC-1/cGM-CSF vaccinated animals but mice vaccinated with TC-1/cGM-CSF and tetanus toxoid combination vaccination generated higher specific lytic activities (R = 2.34, 57.36%) compared with mice vaccinated with tetanus toxoid (R = 1.15, 13%) or TC-1/cGM-CSF (R = 1.81, 44.8%) vaccine alone. The ratio between the percentages of un-pulsed vs E7_49−57_ peptide-pulsed (CFSE^low^/CFSE^high^) was calculated to obtain the numerical valve for cytotoxicity. These data indicate that E7_49−57_ peptide can stimulate CD8+ T cell differentiation into CTL effector cells *in vivo*.

### Combination Vaccination With Tetanus Toxoid and GVAX Induced Enhanced Immuno-Surveillance and Effectively Inhibits Tumorigenesis *in vivo*

**Figure 6:**
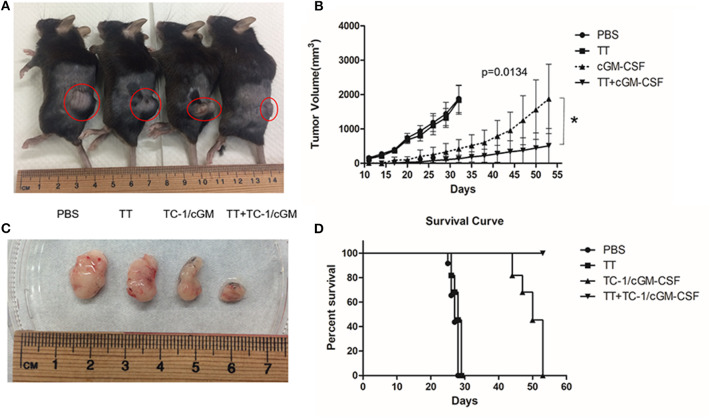
Combination vaccination with tetanus toxoid and GVAX can effectively inhibit tumor growth. **(A)** C57BL/6 mice (*n* = 5) were injected with tetanus toxoid or GVAX or combination of both (4 × 10^6^) subcutaneously at the dorsal flank. Seven days after the last immunization, the mice were inoculated with 2 × 10^5^ TC-1 cells subcutaneously at the right flank. Representative mice bearing xenograft TC-1 tumor. **(B)** Comparison of tumors excised from xenograft mice **(C)**. The tumor size was monitored weekly twice for 7 weeks. The line graph depicts tumor volumes over time in various vaccinated mouse groups. **(D)** Survival curve with mice (*n* = 5) vaccinated with PBS, tetanus toxoid, GVAX and combination of tetanus toxoid and GVAX. The data are presented as the mean ± SD of triplicate values. The log rank (Mantel-Cox) test was used to compare the survival rates among various groups. *p < 0.05; single-classification ANOVA.

To evaluate the anti-tumor efficacy of combination vaccination with tetanus toxoid and GVAX on tumor growth, mice were pre-conditioned twice with tetanus toxoid at two-week intervals followed by vaccination with GVAX at two-week time intervals. The mice were challenged with 2 × 10^5^ TC-1 tumor cells 7 days after the final vaccination (Figure A1). The tumor volume was measured weekly thrice. We observed an inhibition of subcutaneous tumor growth 10 days after the tumor challenge in the group of mice vaccinated with combination vaccination (Figures 6A–C). Group of mice vaccinated with either tetanus toxoid or GVAX alone revealed rapid subcutaneous tumor growth. We observed that group of mice vaccinated with combination vaccination showed TC-1 tumor regression in 90% of the mice, and these mice remained tumor free over the test period. We also observed an increase in the life span for over 53 days in the mice vaccinated with combination vaccination of tetanus toxoid and GVAX compared with those of the group of mice vaccinated with either tetanus toxoid or TC-1/cGM-CSF tumor cell-based vaccine (Figure 6D). These results showed that mice vaccinated with the combination of tetanus toxoid and GVAX displayed the most efficient TC-1 tumor regression compared with mice that had been vaccinated with either tetanus toxoid or GVAX alone.

## Discussion

This study shows that in a cervical cancer mouse model vaccination with a combination vaccine such as tetanus toxoid and TC-1/cGM-CSF (GVAX) regressed tumor growth and enhanced the overall survival of mice compared with mice receiving either tetanus toxoid or GVAX alone. We also found that combination vaccination of tetanus toxoid and GVAX can enhance Th1 and Th2 cell cytokine response and generate memory T cells. Finally, we showed that combination vaccination with tetanus toxoid and GVAX can generate higher percentage of cytotoxic effector T cells *in vivo* compared with mice vaccinated with tetanus toxoid or GVAX alone.

Attenuated bacterial antigens play an important role in immunotherapy as they regulate various immune cells such as tumor-associated macrophages, dendritic cells, tumor-associated lymphoid cells such as NK cells, CD4+, and CD8+ cells and regulatory T cells (39, 40). A recent study in a Glioblastoma tumor model showed that pre-conditioning mice with tetanus toxoid followed by dendritic cell-based vaccination pulsed with Cytomegalovirus phosphoprotein 65 (pp65) can enhance dendritic cell migration bilaterally and significantly improved overall survival (27). These studies showed the significance of bacterial antigens in the field of cancer immunotherapy.

Pro-inflammatory cytokines such as IFN-γ and TNF-α can help stimulate effector cells and enhance tumor cell recognition of cytotoxic effector cells (41, 42). Consistent with this knowledge, our study shows that combination vaccination with tetanus toxoid and GVAX can induce higher level of IFN-γ and TNF-α compared with the group of mice vaccinated with either tetanus toxoid or TC-1/cGM-CSF vaccine alone. We also observed a higher percentage of cytotoxic effector cells in the group of mice vaccinated with the combination vaccination compared with that of the control *in vivo*.

Surprisingly we found a variation in the size of spleens in mice vaccinated with different vaccines and among them mice vaccinated with a combination of tetanus toxoid and GVAX exhibit a higher spleen size compared with the group of mice vaccinated with either tetanus toxoid or GVAX alone. Studies have shown that bigger spleen sizes might be due to higher proliferation rates of the splenocytes after vaccination. Lymphocytes proliferate when it comes in contact with antigen-presenting cells containing peptide-MHC complexes. Generally, splenic populations trap and remove blood antigens and initiate innate and adaptive immune responses against pathogens.

In summary, we demonstrated that combination vaccination with tetanus toxoid and GVAX can elicit a greater anti-tumor immune response in mice compared with those of mice receiving the tetanus toxoid or TC-1/cGM-CSF vaccine alone. In addition, combination vaccination induced long-lasting memory T cell responses of cytolytic effector T cells compared with the control groups. Hence, pre-conditioning with tetanus toxoid prior to vaccination with a tumor-cell based vaccine overexpressing GM-CSF might be an effective strategy to target E7-specific HPV-associated cervical malignancy.

In conclusion, bacterial based antigens such as tetanus toxoid in combination with tumor antigen can be used effectively to enhance the anti-tumor effects and immune-stimulatory capacity of the host infected with HPV. Although using a bacterial-based antigen for immunotherapy has few drawbacks, the promising results of this therapy must be studied extensively to prove its importance in the field of cancer immunotherapy. In this way, the options available for the cancer patients preferring for immunotherapeutic treatment widely opens up. If proven with strong results in the pre-clinical and clinical studies, HPV infected individuals can opt for bacterial immunotherapy in situations where treatment with other immunotherapeutic technique is a problem.

## Data Availability Statement

All datasets generated for this study are included in the article/Supplementary Material.

## Ethics Statement

The animal studies were reviewed and approved by the Review Board of Ethics Committee of Chang Gung University. The statement of approval from the Ethics Committee contains the ethical code CGU107-288 with the date 1st August, 2019.

## Author Contributions

JQ and DA designed experiments, analyzed data, and discussed the data. DA, B-XY, and KS performed the experiments. DA and SS wrote the manuscript and revised the manuscript.

## Conflict of Interest

The authors declare that the research was conducted in the absence of any commercial or financial relationships that could be construed as a potential conflict of interest.
