# Supplementary material for: Combination Vaccination With Tetanus Toxoid and Enhanced Tumor-Cell Based Vaccine Against Cervical Cancer in a Mouse Model
Source: Front Immunol. 2020 May 27;11:927. doi: 10.3389/fimmu.2020.00927 (PMC7269150; doi:10.3389/fimmu.2020.00927)
Supplement: Supplementary file 1 [file Table_1.DOCX]

**Appendix A1**

**Figure 1.** Mice immunization schedule. Mice (n = 5 to 10) vaccinated intramuscularly and subcutaneously with tetanus toxoid and GVAX. Seven days after the last vaccination spleens from the mice were collected, cultured and the medium was collected and analyzed by ELISA for the presence of IFN-γ, TNF-α and IL-4. 30 days after the tumor cell inoculation, spleens and tumors from the mice were collected and analyzed by flow cytometry for CD4+, CD8+, CD44+ memory T cells.
